# The transition from primary colorectal cancer to isolated peritoneal malignancy is associated with an increased tumour mutational burden

**DOI:** 10.1038/s41598-020-75844-6

**Published:** 2020-11-03

**Authors:** Sally Hallam, Joanne Stockton, Claire Bryer, Celina Whalley, Valerie Pestinger, Haney Youssef, Andrew D. Beggs

**Affiliations:** Surgical Research Laboratory, Institute of Cancer and Genomic Science, University of Birmingham, Birmingham, B15 2TT UK

**Keywords:** Predictive markers, Cancer genomics, Gastrointestinal cancer, Colorectal cancer

## Abstract

Colorectal Peritoneal metastases (CPM) develop in 15% of colorectal cancers. Cytoreductive surgery and heated intraperitoneal chemotherapy (CRS & HIPEC) is the current standard of care in selected patients with limited resectable CPM. Despite selection using known prognostic factors survival is varied and morbidity and mortality are relatively high. There is a need to improve patient selection and a paucity of research concerning the biology of isolated CPM. We aimed to determine the biology associated with transition from primary CRC to CPM and of patients with CPM not responding to treatment with CRS & HIPEC, to identify those suitable for treatment with CRS & HIPEC and to identify targets for existing repurposed or novel treatment strategies. A cohort of patients with CPM treated with CRS & HIPEC was recruited and divided according to prognosis. Molecular profiling of the transcriptome (n = 25), epigenome (n = 24) and genome (n = 21) of CPM and matched primary CRC was performed. CPM were characterised by frequent Wnt/ β catenin negative regulator mutations, TET2 mutations, mismatch repair mutations and high tumour mutational burden. Here we show the molecular features associated with CPM development and associated with not responding to CRS & HIPEC. Potential applications include improving patient selection for treatment with CRS & HIPEC and in future research into novel and personalised treatments targeting the molecular features identified here.

## Background

Little is known about the biology of isolated colorectal peritoneal metastasis (CPM), which although a relatively rare phenomenon is one with a high mortality rate^1^. Understanding tumour biology may identify which patients with primary colorectal cancer (CRC) are at risk of developing CPM, and which are suitable for treatment with cytoreductive surgery and heated intra-peritoneal chemotherapy (CRS & HIPEC). CRS & HIPEC (usually using an agent such as mitomycin C or more recently, oxaliplatin) aims to achieve macroscopic tumour resection with multiple visceral and peritoneal resections and ablation of microscopic disease. Five-year survival however varies widely, and morbidity and mortality are relatively high^2^. There is a need therefore to improve patient selection, allowing alternative existing or novel treatment strategies to be used for patients unlikely to respond.

Primary CRC research has identified markers of response to specific treatments, for example KRAS mutation in selection for anti-EGFR mAb therapy^3^. Gene expression signatures have been developed and are in clinical use for prognostication and therapeutic stratification in breast cancer^4–7^. Gene expression profiling in primary CRC has identified signatures associated with the development of metastasis^6^. One small study combining a small number of CPM with a larger cohort of appendix adenocarcinoma identified a signature predictive of reduced overall survival (OS) following CRS & HIPEC; these are however two biologically distinct tumours, appendix having significantly improved prognosis^7^.

The dysregulation of methylation is a key step in tumorigenesis CpG island promoter methylation (CIMP) appears to be stable between matched primary CRC and hepatic metastasis suggesting an epigenetic methylation programme is established prior to the development of metastasis^8–10^. Hypermethylation of KRAS, Wnt modulators, tumour suppressor genes, CIMP and hypomethylation of oncogenes are associated with an unfavourable response to chemotherapy and anti-EGFR antibodies as well as tumour recurrence and reduced OS in primary and metastatic CRC^11–16^. Chromosomal instability is ubiquitous in cancer, increased copy number alteration, indicative of chromosomal instability is found in metastatic CRC^17,18^. Lopez-Garcia et al.^19^ demonstrated that the evolution of chromosomal instability is depending on cellular tolerance, either via dysregulation of TP53 or via alternate escape mechanisms such as dysfunction of BCL9L regulated caspase signalling.

CRC metastatic drivers are less clearly defined, apart from TP53 which is well characterised as being present in metastatic cancer^20^. Some studies have found mutations exclusive to metastatic sites^21,22^, whereas others found similar patterns of mutation between primary and metastasis^23^. Studies have examined the somatic mutations in CPM and their prognostic implications. These studies are limited to individual or small panels of mutations routinely tested for in clinical practice with limited evidence to suggest which genes should be included in panel sequencing in CPM. Schneider et al. examined the *KRAS* and *BRAF* mutation status of patients with CPM who underwent CRS & HIPEC^24^. They found mutations of *RAS/RAF* were associated with reduced OS independent of the use of targeted anti-EGFR treatment^24^. Sasaki et al. examined the *KRAS, BRAF* and *PIK3CA* mutation status of patients with metastatic CRC, with or without CPM^25^. They found the incidence of *BRAF* mutation was significantly associated with the presence of CPM but not with prognosis^25^.

The landscape of metastatic colorectal cancer was studied by the MSK-IMPACT^20^ group which undertook panel based sequencing of 1134 metastatic colorectal cancers. Of these 39 patients were defined as “peritoneal” malignancy, it is unclear whether these were isolated peritoneal metastasis. Only 14 of these patients had metasectomy. 7 of these had peritonectomy suggesting isolated disease suitable for resection. These tumours were also not studied with matched primary tumour of origin.

There is a need to improve the outcomes for patients with CPM and significant variation in survival despite patient selection for treatment using known prognostic factors. There is a paucity of knowledge concerning CPM tumour biology. Understanding tumour biology will identify patients with primary CRC at risk of developing CPM, those suitable for treatment with CRS & HIPEC or alternative existing and novel treatment strategies. This study aims to determine the landscape of gene expression, methylation, and somatic mutation profile associated with the transition from primary CRC to isolated CPM and determine the association between these and prognosis following CRS & HIPEC in order to identify therapeutic targets.

## Methods

### Patient cohorts

This study obtained ethical approval from the North West Haydock Research Ethics Committee, (15/NW/0079), project ID (17/283). Participants gave informed consent. All experiments were performed in accordance with relevant guidelines and regulations Consecutive retrospective patients were recruited from an internally held database of all patients undergoing CRS & HIPEC at Good Hope hospital from 2011 to 2017. Patients with CPM (adenocarcinoma), no extra-abdominal metastasis, a complete resection (CC0) and a peritoneal carcinomatosis index (PCI) of < 12 were eligible for inclusion. The completeness of cytoreduction score describes the degree of macroscopic tumour remaining after CRS and the likelihood of benefit from intraperitoneal chemotherapy^26^. Patients with no residual tumour score CC0, residual tumour < 0.25 cm, CC1, residual tumour 0.25–2.5 cm CC2. The extent of peritoneal metastasis is described by the PCI score. A PCI of ≥ 12 is poor prognostic factor for patients undergoing CRS & HIPEC^27^. Patients were divided into two groups. CRS & HIPEC is a long operation associated with a protracted inpatient and high dependency (HDU) or intensive care (ITU) stay an associated mortality of 1–12% and morbidity of 7–63% and a prolonged post-operative recovery^28–37^.With palliative chemotherapy DFS is 11–13 months and therefore patients post-treatment (CRS & HIPEC) with disease free survival (DFS) < 12 months were defined as “non-responders”^38^. Patients undergoing therapy with DFS > 12 months were defined as “responders”. Patients were imaged with CT which was reported by an experienced CPM radiologist, diagnostic laparoscopy was not used, not all patients with recurrence are suitable for iterative CRS & HIPEC and so this is not a standard procedure in their follow up. Adhesions following primary excision and CRS & HIPEC may also preclude accurate assessment of peritoneal recurrence in all areas with laparoscopy. Disease recurrence was determined when confirmed by CT and MDT review.

Demographic, tumour and treatment details were compared between the prognostic cohorts. For continuous variables, the students T-test was applied to normally distributed data and Mann Whitney-U to non-normally distributed data. Categorical variables were compared with the Chi-squared test or Fishers exact test. A *p* value of < 0.05 was considered statistically significant. DFS survival between the responders and non-responders was compared using the Kaplan Meier method. Statistical analysis was performed in IBM SPSS Statistics for Windows, Version 24.0^39^.

### Nucleic acid extraction

DNA and RNA were extracted from histologically confirmed Formalin fixed, paraffin embedded (FFPE) scrolls using the Covaris E220 evolution focused-ultrasonicator and the truTRAC FFPE total NA Kit. All peritoneal metastases samples were taken at the commencement of surgery. Nucleic acid concentration was quantified using the Qubit 3.0 Fluorometer and Qubit RNA / DNA HS (high sensitivity) assay kit. Nucleic acid quality was measured by electrophoresis using the Agilent 2200 TapeStation Nucleic Acid System, Agilent 2200 TapeStation Software A.01.05 and the Aligent High Sensitivity RNA / DNA ScreenTape and reagents.

### RNA library preparation, sequencing and bioinformatics

RNA library preparation was performed using the Lexogen Quant Seq 3′ mRNA-Seq Library Prep kit. RNA libraries were denatured, diluted, loaded onto a 75-cycle High output flow cell and sequenced using the NextSeq500 at 2.5–5 million reads^40^.

Quality control, trimming and alignment to the reference genome, (NCBI build 37, hg19) was performed with the Partek Flow genomics suite software package (Partek, St Louis, MI, USA). The gene expression profiles of primary and CPM and responders and non-responders were compared using gene Specific Analysis (GSA) Modelling using Partek flow with a false discovery rate (FDR) of < 0.1. Gene specific enrichment analysis (GSEA) and gene expression pathway analysis was performed using Partek flow, a *p* value of ≤ 0.05 was considered statistically significant.

CMS and CRIS classifications were performed using ‘CMScaller’ (v0.99.1) in the R package, version 2.10.2^38,41,42^. Fishers exact test was used to compare contingency between primary and CPM and responders and non-responders in IBM SPSS Statistics for Windows, Version 24.0^39^. A *p* value of < 0.05 was considered significant.

### Methylation array and bioinformatics

DNA was treated with sodium bisulphite using the Zymo EZ-DNA methylation kit, according to manufacturer’s instructions. Degraded FFPE DNA was restored prior to methylation array with the Infinium HD FFPE restore kit, according to manufacturer’s instructions. Methylation array was performed according to the Infinium MethylationEPIC BeadChip Kit manufacturer’s instructions. BeadChips were imaged using the Illumina iScan system. Initial data quality was checked using GenomeStudio Methylation Module Software.

Raw data was loaded into the RStudio version 3.5.0 software using the minifi package. Bioinformatics analysis was performed using the Chip Analysis Methylation Pipeline (ChAMP) R package, version 2.10.2^43,44^. Probes with signals from less than three functional beads, low confidence with a detection *p* value > 0.01, covering SNPs, non-CpG and those located on the X and Y chromosome where filtered. Beta-mixture quantile normalization (BMIQ) was applied and a singular value decomposition (SVD) performed to identify batch effects. The association between methylation and prognosis was determined using the Bioconductor R package limma and bumphunter functions. Copy number alteration calling was performed using the CHAMP CNA function with a significance threshold of, *p* value < *p* < × 10^–10^.

### Exome capture, high-throughput sequencing and bioinformatics

DNA was sheared using the Covaris E220 evolution focused-ultrasonicator to produce a uniform 150 bp fragment size. Libraries were prepared using the TruSeq Exome Kit then denatured, diluted, loaded onto a 150-cycle High output flow cell and sequenced using the NextSeq500.

Sequencing reads were assessed using FastQC. Sequences with a Phred score of < 30 were removed giving a base call accuracy of 99.9%. Sequence reads were aligned to the human reference genome, (hg19) using the Burrows–Wheeler Aligner (BWA) package^45^. SAMTools was used to generate chromosomal coordinate-sorted BAM files and Picard was used to remove PCR duplicates^46^. Somatic variants were called from matched tumour-normal samples using Strelka2 in tumour/normal mode^47^. Somatic variants were viewed, filtered and annotated in genomics workbench^48^. Mutations with a MAF of > 1% in known variant databases, (dbSNP and 100,000 genomes) were filtered. Mutations were annotated with information from known variant databases, (dbSNP and 100,000 genomes), PhastCons score and functional consequences. The prognostic groups were compared using Fischer exact test to identify potential candidate driver mutations for non-responders. Somatic mutations were entered into the IntOGen platform for further analysis^49^. The IntOGen-mutation platform incorporates a number of pipelines to identify cancer driver mutations and activated pathways^49^. The OncodriveFM pipeline identifies mutations with a high functional impact using three scoring methods (Sorting Intolerant From Tolerant, (SIFT)^50^, PolyPhen2^51^, and Mutation Assessor scores)^49,52^, and assesses the likelihood that such mutations are cancer drivers. The OncodriveCLUST pipeline assesses the clustering of mutations to identify relevant activated pathways^49^. MSI assessment was carried out using MSI_classifier_v3 (https://rpubs.com/sigven/msi_classification_v3).

### Ethics approval and consent to participate

North West Haydock Research Ethics Committee, (15/NW/0079), project ID (17/283).

## Results

### Patient cohort

From 2011 to 2017 a total of n = 161 patients underwent CRS & HIPEC at University Hospitals Birmingham, n = 88 patients for metachronous CPM.

Patients were excluded for the following reasons: other primary tumour (appendix, pseudomyxoma peritonei, ovarian) n = 49, synchronous colorectal cancer n = 26, no primary tumour available n = 53 CC2 resection n = 8^26^, PCI of ≥ 12 n = 20, follow up period of ≤ 12 months n = 27, leaving n = 28 patients. Complete information regarding the primary CRC pathology and treatment was available for n = 26 patients who form the basis of this study. Each patient had matched normal, primary CRC and CPM samples.

Thirteen patients had a DFS of 24 months (15–72 range) following CRS & HIPEC and formed the ‘responders cohort, thirteen patients had a DFS of 6 months (2–11 range) and formed the ‘non-responders’. There were no significant differences between cohorts in demographics, primary CRC or CPM tumour, treatment or follow up (Table 1). No patients had neoadjuvant therapy for their primary tumour. Three patients (all in the responders group) had poorly differentiated, mucinous adenocarcinoma, one had signet ring adenocarcinoma (in the non-responders group) and all the others had moderately differentiated adenocarcinoma.

**Table 1:** Comparison of responders and non-responders to CRS & HIPEC.

		Responders	Non-responders	*p* Value
Age, mean + /−SD		58 ± 13	58 ± 9	0.97
Gender, male		n = 7 (54)	n = 7 (54)	0.68
Tumour location	Right	n = 9 (69)	n = 6 (46)	
	Transverse	n = 1 (8)	n = 0 (0)	
	Left	n = 3 (23)	n = 7 (54)	0.33
T stage primary	3	n = 3 (23)	n = 3 (23)	
	4a	n = 5 (38.5)	n = 7 (54	
	4b	n = 5 (38.5)	n = 3 (23)	0.66
N stage primary	0	n = 4 (31)	n = 1 (8)	
	1	n = 7 (54)	n = 5 (38)	
	2	n = 2 (15)	n = 7 (54)	0.86
DFI months		25 ± 9	24 ± 12	0.83
PCI score, median (range)		5 (3–12)	8 (2–12)	0.019
CC score	CC0	n = 13 (100)	n = 13 (100)	1
	CC1	n = 0 (0)	n = 0 (0)	
	CC2	n = 0 (0)	n = 0 (0)	
Follow up, months, median (range)		29 (19–72)	16 (5–55)	0.11
Adjuvant treatment	Yes	n = 11 (85)	n = 12 (92)	0.38
	No	n = 2 (15)	n = 1 (8)	
DFS, median (range)		24 (15–72)	6 (2–11)	< 0.0001
OS, median (range)		29 (19–72)	16 (5–55)	0.12

*N* number value in parenthesis, percentage, *DFI* disease free interval, time from primary CRC to metachronous CPM, *PCI* peritoneal carcinomatosis index, *CC score* completeness of cytoreduction, *DFS* disease free survival, *OS* overall survival.

Log rank *p* < 0.0001.

Following nucleic acid extraction all patients had adequate CPM RNA for RNAseq (n = 13 responders, n = 13 non-responders), n = 25 had matched primary CRC samples. For methylation array n = 24 patients (n = 12 responders, n = 12 non-responders) had adequate DNA. As the Infinium methylation array comprises a 32-prep kit, n = 4 responders and n = 4 non-responders primary tumours were matched to these. For exome sequencing n = 24 patients (n = 12 responders, n = 12 non-responders) had adequate DNA from both the primary and CPM samples, extraction of DNA from normal tissue resulted in n = 21 samples (n = 9 responders, n = 12 non-responders).

### Exome sequencing

Across all six sequencing runs, we obtained a median of 60X coverage (42–166) with a median uniformity of 88% (71–89).

### Somatic mutations identified in the primary and matched CPM cohort

In the matched CPM cohort, a total of n = 244,531 somatic SNV’s were identified (CPM-primary subtraction) significantly more than found in the matched primary cohort (n = 112,420).

**Figure 1:**
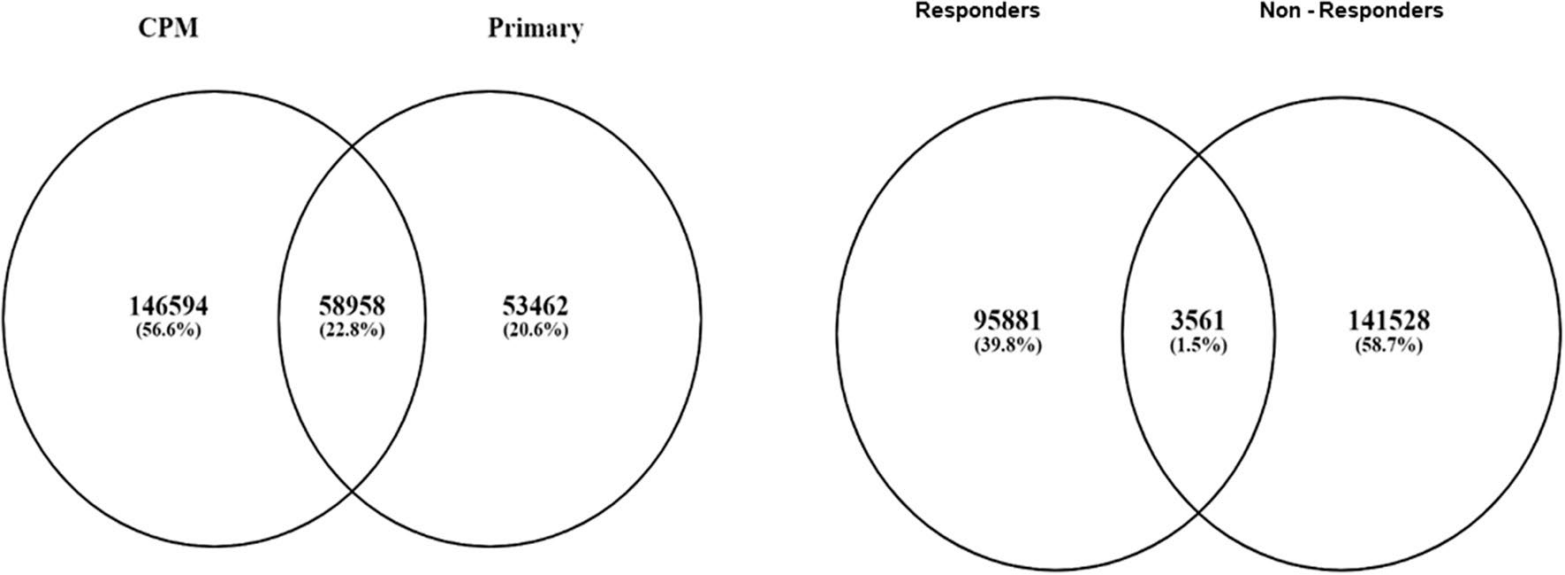
Venn diagrams depicting the frequency of mutations exclusive to and shared between primary CRC and matched CPM and responders and non-responders.

Nine CPM samples, 9/24 (56%) had a high tumour mutational burden TMB ≥ 10 mut/Mb^53^ compared with 7/24 (30%) samples in the matched primary cohort. Mutations were identified in n = 69 of n = 95 known CRC driver genes, n = 51 were shared between the primary and CPM, n = 13 were novel (supplementary table S1)^54^. Of the somatic variants identified in CPM, n = 58,958 (29%) were present in the primary CRC, n = 205,552 variants occurred exclusively in the CPM suggesting a significant accumulation of mutations in the transition to CPM (Fig. 1). OncodriveFM identified n = 265 potential driver genes with high levels of functional mutation (Q-value < 0.05) in the CPM cohort: *FLNB*, *SPTB, PPL, TP53, PDE4DIP*, *RIOK2, CDC16, NUP98, CDC16* and *SVEP1* (supplementary table S2), however these results must be treated with caution due to the bias of the hypermutator phenotype. KEGG pathway analysis of mutations demonstrated enrichment in pathways concerning the immune system, signalling, metabolism and cancer (supplementary table S1). In the CPM group KRAS or BRAF status was not significantly associated with prognosis (chi2 *p* = 1.00).

Clonality analysis with SuperFreq showed significant (Wilcoxon rank *p* = 0.007) differences between the responders and non-responders groups, with a median of 2 clones in the responders group of primary tumours (range 1–4) and 3 clones in the non-responders group (range 2–7). In the peritoneal metastases there were a median of 3 clones in both the responders (range 1–4) and non-responders (range 2–5) groups. Of note, in the non-responders group during clonal expansion, the dominant clone in the peritoneal metastasis group arose de-novo rather than being a prior clone that existed in the primary tumour (Supplementary Fig. 1, S1e primary tumours, 9/21 were MSI (47.4%) and 10/21 were MSS (52.6%) whereas in the isolated peritoneal metastasis group, 4/21 (19.0%) were MSS and 17/21 MSI (81.0%) Demonstrating that there was a significantly higher rate of MSI in the isolated peritoneal metastasis group (*p* < 0.05, Chi2).

Non-responders had a higher frequency of somatic mutations: 60% of all mutations in CPM cohort vs. 40%. Non-responders more commonly had a high tumour mutational burden, TMB ≥ 10 mut/Mb^53^, 56% vs. 44%. Of the somatic mutations identified in non-responders, n = 35,461 (30%) were present in responders, n = 145,089 variants occurred exclusively in non-responders, suggesting a high tumour mutational burden was associated with non-response to CRS & HIPEC (Fig. 1). Mutational signature analysis of the MSI tumours demonstrated a predominance of signature 5 (associated with mutational “clock” effects), signature 26 (associated with defective mismatch repair) and signature 20 (associated with defective mismatch repair).

Comparison of somatic mutations in responders and non-responders identified two potential candidate genes to identify non-responders, *FAM13A* and *PIEZO2* (Fishers exact *p* < 0.05, FDR = 0.53) (Table 2).

**Table 2:** Potential candidate variants, non-responders to CRS & HIPEC.

Chr	Position	Reference	Allele	*p* Value	FDR	Sample frequency (case)	Sample frequency (control)	Gene ID
4	93,084,410	C	G	0.007	0.53	62.5	0	*FAM13A*
18	11,552,313	G	C	0.023	0.53	50	0	*PIEZO2*

CPM identified through Fisher exact test, genomics workbench (Chr, chromosome, FDR, false discovery rate).

### Differentialene expression

#### Differential gene expression between primary CRC and matched CPM

**Figure 2:**
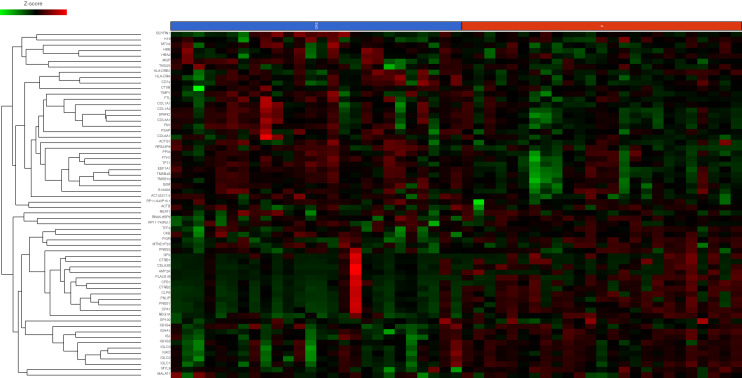
Heatmap of differential gene expression in 100 highest genes ranked by variance between primary CRC (P, red) and colorectal peritoneal metastasis (CRS, blue). Sample type is indicated at the X axis of the heatmap with individual genes on the Y-axis. Individual IDs of each patient are below the indicators of primary or CRS sample. Gene expression as indicated by the Z-score is displayed as colour ranging from green to black to red as shown in the legend. Created in Partek Flow.

Primary CRC and matched CPM showed differential expression of n = 65 genes with an FDR < 0.1. (Fig. 2) Sixteen genes showed significantly decreased expression in CPM compared with primary CRC (Table 3). Forty-nine genes showed significantly increased expression in CPM compared with primary CRC (Table 3). A KEGG pathway analysis was performed to identify the enriched biological functions among the differentially expressed genes (Supplementary Table 1). The expression of *FABP6*, an intercellular bile acid transporter, was decreased 34.30-fold in CPM. *OLFM4* is a target of the Wnt/β-catenin pathway, its expression was reduced 3.77-fold in CPM. *DCN* and PTEN are able to initiate a number of signalling pathways including *ERK* and *EGFR* leading to growth suppression, their expression was increased 3.3-fold and 3.25 fold in CPM, this was unexpected and in contrast to the literature^55^. *NF-κBIA* expression was increased 3.24-fold in CPM, its upregulation may reflect increased *NF-κB* activity in the development of CPM^56^.

**Table 3:** The top 10 genes with significantly altered expression (FDR < 0.1) in CPM samples compared with primary CRC samples.

Rank	Gene name	Function	Fold change	FDR *p* value
**Reduced expression CPM samples vs. primary CRC**				
1	FABP6	Intracellular bile acid transporter	− 34.30	1.74 × 10^–06^
2	DEFA6	Cytotoxic peptide involved in host intestine defence	− 8.15	8.55 × 10^–06^
3	DMBT1	Tumour suppressor	− 6.06	2.43 × 10^–04^
4	TTC38	Protein coding gene	− 4.56	5.80 × 10^–05^
5	OLFM4	Wnt/β-catenin pathway target	− 3.77	1.01 × 10^–04^
6	IGHA1	Immune receptor	− 3.66	4.23 × 10^–05^
7	CES2	Intestinal enzyme controlling drug clearance	− 3.20	6.84 × 10^–05^
8	NDUFS6	Enzyme in electron transport chain of mitochondria	− 2.70	7.74 × 10^–05^
9	P2RY11	G-protein coupled receptor	− 2.53	6.37 × 10^–04^
10	MUC2	Encodes a mucinous intestinal coating	− 2.34	7.22 × 10^–04^
**Increased expression CPM samples vs. primary CRC**				
1	CD53	Tetraspanin	7.29	5.87 × 10^–05^
2	CYR61	Extracellular signalling protein	4.24	3.12 × 10^–04^
3	CXCL12	G-protein coupled receptor	3.64	9.25 × 10^–04^
4	NR2F1	Nuclear hormone receptor and transcriptional regulator	3.53	7.09 × 10^–04^
5	CTGF	Connective tissue growth factor	3.49	1.55 × 10^–04^
6	CSTB	Cystatin	3.41	6.13 × 10^–04^
7	TSC22D3	Anti-inflammatory protein glucocorticoid (GC)-induced leucine zipper	3.36	3.94 × 10^–04^
8	DCN	Tumour suppressor gene	3.30	6.19 × 10^–05^
9	PTEN	Tumour suppressor gene	3.25	9.28 × 10^–04^
10	NF-κBIA	Inhibits the *NF-κB* transcription factor	3.24	1.06 × 10^–04^

Gene specific enrichment analysis (GSEA) results are presented in supplementary table 5 We identified 848 upregulated gene ontology categories in CPM and 14 upregulated gene pathways. which may contribute to the pathogenesis of CPM: the mTOR pathway as well as immune pathways including the intestinal immune network for IgA production, Leukocyte transendothelial migration and the actin cytoskeleton pathway.

#### Differential gene expression between non-responders and responders to CRS & HIPEC

**Figure 3:**
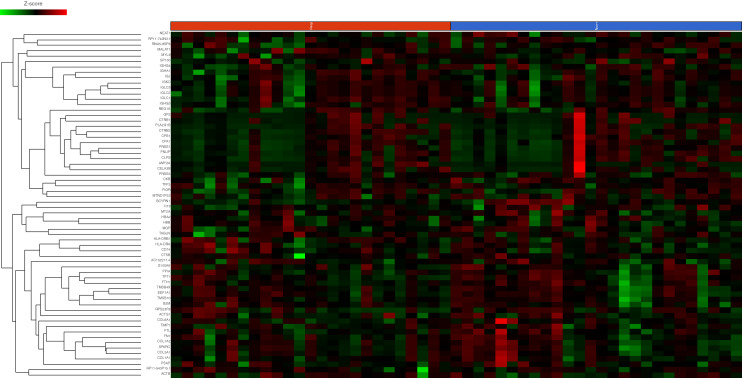
Heatmap differential gene expression of top 100 genes as ranked by variance between responders (blue) and non-responders (red)Sample type is indicated at the transverse border of the heatmap with individual genes on the longitudinal border. Gene expression as indicated by the Z-score is displayed as colour ranging from green to black to red as shown in the legend. Created in Partek Flow.

One hundred and forty-nine genes showed increased expression in non-responders (Fig. 3). Five genes showed decreased expression in non-responders, however none had a fold change ≥ 1.5 suggesting minimal difference in expression between the responders and non-responders (Supplementary Table 2). KEGG pathway analysis demonstrated enrichment in endocytosis, metabolism, phagocytosis, cell movement and architecture, bacterial and viral cell infection, transcription and the expression of genes controlling apoptosis, cell cycle, oxidative stress resistance and longevity (Table 3). The expression of *CEACAM1,* a member of the carcinoembryonic antigen (*CEA*) immunoglobulin family, was increased 8.27-fold in non-responders^57^.

*AXIN1* encodes a cytoplasmic protein which forms the ß-Catenin destruction complex, a negative regulator of the WNT signalling pathway^58^. *AXIN1* expression was increased 5.42-fold in non-responders^59^.

Gene specific enrichment analysis (GSEA) results are presented in supplementary table 6. We identified 591 upregulated gene ontology categories in CPM and 15 upregulated gene pathways. which may contribute to the pathogenesis of CPM: Endocytosis, the adherens junction pathway and immune pathways such as those regulating the bacterial invasion of epithelial cells.

**Figure 4:**
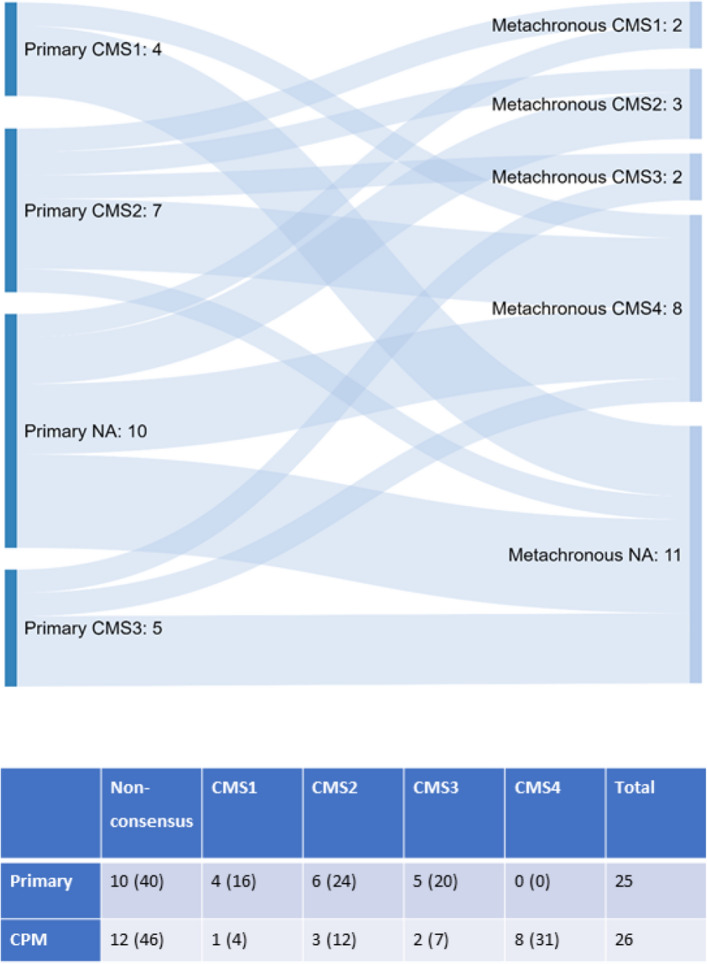
Sankey diagram depicting the transition in consensus molecular subtypes (CMS) from primary to CPM. CMS classifications were performed using ‘CMScaller’ (v0.99.1) in the R /Bioconductor statistics package. Classifications include CMS1 to CMS4, non-consensus samples do not have a consistent pattern of subtype label association. Primary CRC samples, classification and number are shown to the left of the diagram with CPM samples, classification and number to the right of the diagram. Fishers exact *p* value 0.085, values in parenthesis percentages.

Amongst the n = 51 primary CRC and CPM samples n = 29 were representative of each CMS subtype, the remaining n = 22 samples did not have a consistent pattern (Fig. 4). Comparison of the CMS subtypes in primary and CPM and prognostic groups revealed an apparent transition from primary CRC to CPM. No primary CRC samples were classified as CMS4 (mesenchymal subtype characterized by prominent transforming growth factor activation, stromal invasion and angiogenesis) compared to 31% of CPM (*p* = 0.085). Secondly, non-responders were more commonly CMS4, 46% vs. 15% (*p* = 0.005, Table 4).

**Table 4:** CMS classification responders vs. non-responders to CRS & HIPEC.

	Non-consensus	CMS1	CMS2	CMS3	CMS4		Total
Responders	10 (77)	0 (0)	0 (0)	1 (8)	2 (15)		13
Non-responders	2 (15)	1 (8)	3 (23)	1 (8)	6 (46)		13

CMS Fishers exact *p* value 0.005, CRIS Fischer’s exact *p* value 0.148, values in parenthesis percentages.

### Methylation

#### Differential methylation between primary CRC and matched CPM

Thirty-two samples in total were hybridised successfully to the Illumina HumanMethylation EPIC microarrays. DMPs were called between the primary CRC and CPM. The top ranked differentially methylated probe was cg04146982, BF 34.5, adjusted *p* value 5.67 × 10^–16^ (chr8:144,943,810–144,943,810, hg19 coordinates), which tags a CpG dinucleotide 3651 bp upstream of the transcription start site of gene Epiplakin 1, (EPPK1)^60^. EPPK1 is part of the Plakin family an important component of the cell cytoskeleton^61^. The other DMP was cg12209861, BF 7.1, adjusted *p* value 0.059 (chr4:37,459,078–37,459,078, hg19 coordinates), 3526 bp upstream of the transcription start site of gene Chromosome 4 Open Reading Frame 19, (*C4orf19*). DMRs were called between primary CRC and CPM via the dmrLasso function of the CHAMP pipeline (Supplementary Table 3). The top 10 most DMRs were in the region of *IGF2*, *ZNF461*, *RASGFR1, CELF4*, *ZSCAN18*, *EDNRB*, *ZBED9, VTRNA2-1*, *ZNF256 and EGFLAM.* KEGG pathway analysis did not reveal any significantly enriched pathways.

Comparison of CNA between primary and CPM via methylation arrays did not identify and significant differences in CNA between primary and CPM at a stringent *p* value of < × 10^–10^ however a number of CNA were identified at a lower significance threshold, *p* = 2.78 × 10^–07^ (Supplementary Table 4).Genes showing CNA gains of known significance in patients with CPM included; *TRIM3, 5, 6, 21 and 22, MT1A, 2A, 3, 4* encode proteins of the metallothionein family.

#### Differential methylation between non-responders and responders to CRS & HIPEC

The top ranked differentially methylated probe was cg07951355, BF = 6, (chr1:40,123,717) which tags an intergenic region 1076 bp before gene *NT5C1A*. Cg25909064, BF 4 adjusted *p* value 0.47 (chr11:120,081,487–120,082,345) which tags an intron of gene *OAF* and cg12977942, BF 4 adjusted *p* value 0.47 (chr5:92,839,309–92,839,309) which tags an intron of gene *NR2F1-AS1*^60^. Six significant DMRs (Supplementary Table 3) were identified in the regions of *NKX6-2, CHFR, GATA3*, *IRX5*, *HCK* and *BC019904*. KEGG pathway analysis did not reveal any significantly enriched pathways.

Comparison of CNA between the CPM prognostic groups identified recurrent gene losses at chromosomes 3, 4, 14, 15, 17 and 19 (Supplementary Table 4). CNA losses clustered in the RAS-MAPK-ERK signalling pathway suggesting dysregulation in non-responders.

Comparison of CNA between the CPM prognostic groups identified n = 19 gene gains at chromosomes 9, 10 and 11. Genes showing CNA gains in non-responders included: *SIT1, RNF38, MELK, PAX5, SHB, ZEB1, DEAF1, ANTXR, EPS8L2* and *PIDD1.*

## Discussion

This study determined the gene expression, CNA, methylation and somatic mutation profile of primary CRC and matched isolated CPM to determine whether there were changes associated with the development of CPM or predicting prognosis for patients with CPM. To our knowledge, this is the first such analysis in a cohort of patients with isolated CPM suitable for treatment with CRS & HIPEC. The MSKCC cohort of metastatic cancer^20^ had a diverse range of metastatic cancer, none of whom overlapped with the type we have studied, which is isolated colorectal peritoneal metastasis, with matched primary samples, suitable for cytoreduction.

Within this study responders and non-responders to CRS & HIPEC were well matched by demographics, tumour stage, treatment and follow up. PCI varied between groups with responders having a median PCI of 5 (3–12) and non-responders a median PCI of 8 (2–12). A PCI of greater than 12 is associated with reduced survival following CRS & HIPEC, no significant difference is consistently found at PCI levels below this^27^.

Comparison of patients with primary CRC and metachronous CPM identified biological changes associated with the transition from primary CRC to CPM. Hypermethylation, CNA and hypermutation resulted in the inactivation of tumour suppressors and oncogene activation in CPM, (TP53, VTRA2-1, TRIM proteins). These changes suggest a rapid rate of tumour growth unchecked by tumour suppressor or apoptotic mechanisms.

Increased MAPK and Wnt/β-catenin pathway activation was noted in CPM. Gene expression of negative regulators of the Wnt pathway was reduced, (OLFM4, DEAFA6), negative Wnt regulators contained somatic mutations, (APC, RNF43, FAM123B and TSC1), and the MAPK marker, RASFGFR1 was hypermethylated suggesting persistent activation of MAPK and Wnt pathways. Multiple mutations of negative Wnt signalling regulators make this an attractive therapeutic target. Porcupine inhibitors mediate the palmitoylation of Wnt ligands, blocking Wnt signalling. The porcupine inhibitor LGK974 inhibits the upstream negative Wnt regulator mutant RNF43 and is a potential therapeutic target in CPM^62^.

CPM contained a high proportion of MSH6 somatic mutations suggesting deficiency in the mismatch repair pathway and MSI. MSH6 mutations are commonly found in isolated peritoneal metastasis^59^. As expected for tumours with mismatch repair deficiency both the primary CRC and CPM cohort had a high tumour mutational burden, crucially this suggests they may have a good response to treatment with immune checkpoint inhibitors such as pembrolizumab^63^, a new therapeutic avenue for these difficult to treat patients. The frequency of hypermutation seen in our study (48%) was considerably higher than that observed for both the MSKCC metastatic disease cohort (5%) and the TCGA Colorectal^64^ cohort (10%). The expression of genes regulating innate immunity however was downregulated, (DEFA6, DMBT1, MUC2) or altered via somatic mutations, (HLA-A antigen) suggesting immune evasion in the transition to CPM which may reduce the likelihood of successful PD-1 therapy.

The expression of genes supressing invasion, migration and EMT was downregulated or hypermethylated, (MUC2, MMP26, ILK, FLNB, SPTB, PPL, and SVEP1) and those triggering these processes upregulated, (CYR61, CXCL12, CTGF, and CSTB). These changes suggest a mechanism by which CPM cells metastasise from the primary CRC. In keeping with changes in EMT regulators there appeared to be a transition in CMS subtypes towards CMS4 from primary CRC to CPM. The CMS4 subtype is an interesting therapeutic target, *TGFβ* signalling inhibitors and targeted immunotherapies have been trialled with success in pre-clinical models to block cross talk between the tumour microenvironment and halt disease progression of stromal rich CMS4 CRC^65,66^.

Methylation appeared to be dysregulated in CPM with a bias towards a hypermethylator phenotype caused by somatic mutation of the TET2 tumour suppressor and CDH7 chromatin regulator. Active DNA demethylation by TET enzymes is an important tumour suppressor mechanism in a variety of cancers^67–69^. Downregulation of CES2, a gene known to activate the prodrug irinotecan, a chemotherapy used as part of the FOLFIRI regimen in the UK in the adjuvant treatment of primary CRC and CPM was seen in this cohort. Resistance to the treatment of primary CRC may in part explain the development of CPM.

*CEACAM1* expression correlates with metastasis and reduced survival in CRC and was upregulated in this cohort of patients^70^. Novel therapies in the form or CEA TCB IgG-based T-cell bispecific antibodies (Cibisatamab) may therefore be of benefit^71^. Additionally there was a downregulation of gene expression of negative regulators of the Wnt pathway, (AXIN1) and somatic mutations of key Wnt regulators, (FAM13A) and hypermethylation of MAPK and TGF-β pathway markers, (RAB8A, RAB34, FGF5 and BMP3) suggesting persistent activation of MAPK, TGF-β and Wnt in non-responders to CRS & HIPEC.

A recent randomised controlled trial has called into question the use of HIPEC in CPM, PRODIGE-7 treated patients with CPM with CRS & HIPEC or CRS alone in addition to systemic chemotherapy. PRODIGE-7 suggests no added benefit from HIPEC however this study was not powered to stratify the impact of HIPEC according to PCI score, on subgroup analysis patients with a PCI of 11–15 had significantly improved median survival with the addition of HIPEC 41.6 months vs. 32.7 months *p* value 0.0209^72^.

A relative weakness of this study is the small cohort of patients, the biological changes identified here form a starting point in identifying the tumour biology associated with the development of CPM and predicting non-responders to CRS & HIPEC. However, we have identified multiple potential targets for therapy, along with the important finding that CPM appears to be a hypermutated, hypermethylated, immune evasive cancer which allows it to be potentially targeted by emerging novel therapeutics. Our study findings have implications for the recent addition of oxaliplatin to HIPEC, as the FOXTROT study of neoadjuvant therapy in colorectal cancer showed that oxaliplatin has no effect in dMMR tumours.

## Conclusions

Patients with colorectal peritoneal metastasis (CPM) secondary to colorectal cancer have limited survival with the best available treatments. Despite selection for treatment using known prognostic factors survival varies widely and can be difficult to predict. There is a paucity of knowledge concerning the biology of CPM, it is likely that there are additional biological markers of response to currently available as well as novel or re-purposed alternative treatments. Here we have comprehensively profiled a cohort of patients with isolated CPM and identified a number of therapeutically targetable alterations including mutations in Wnt/β catenin regulators (via Porcupine inhibitors), the mismatch repair pathway (via PD-1/CTLA-4 immunotherapy) and methylation regulators. We suggest that these are urgently investigated in a larger cohort with the development of pre-clinical models as, in particular, the finding that these patients may be sensitive to immunotherapy may radically change the therapy options available for this difficult to treat group of patients.

## Supplementary information

Supplementary Information 1.

Supplementary Information 2.

Supplementary Information 3.

## Data Availability

The data that support the findings of this study are available from the corresponding author upon reasonable request.

## Abbreviations

CRC: Colorectal cancer
CPM: Colorectal peritoneal metastasis
CRS & HIPEC: Cytoreductive surgery and heated intraperitoneal chemotherapy
DFS: Disease free survival
DMR: Differentially methylated regions
OS: Overall survival
FFPE: Formalin fixed paraffin embedded
