# Supplementary material for: The transition from primary colorectal cancer to isolated peritoneal malignancy is associated with an increased tumour mutational burden
Source: Sci Rep. 2020 Nov 3;10:18900. doi: 10.1038/s41598-020-75844-6 (PMC7641117; doi:10.1038/s41598-020-75844-6)
Supplement: Supplementary file 1 — Supplementary Information 1. [file 41598_2020_75844_MOESM1_ESM.docx]

**Supplementary materials for:**

**The transition from primary colorectal cancer to isolated peritoneal malignancy is associated with an increased tumour mutational burden**

Sally Hallam^1^, Joanne Stockton^1^, Claire Bryer^1^, Celina Whalley^1^, Valerie Pestinger^1^, Haney Youssef^1^, Andrew D Beggs^1^

1 = Surgical Research Laboratory, Institute of Cancer & Genomic Science, University of Birmingham, B15 2TT.

**Correspondence to:**

Andrew Beggs, [a.beggs@bham.ac.uk](mailto:a.beggs@bham.ac.uk)

**Supplementary tables & figures**

Supplementary figure 1: Clonality river plot of primary tumour, normal germline and colorectal peritoneal metastasis (CPM). Clonality on Y axis.


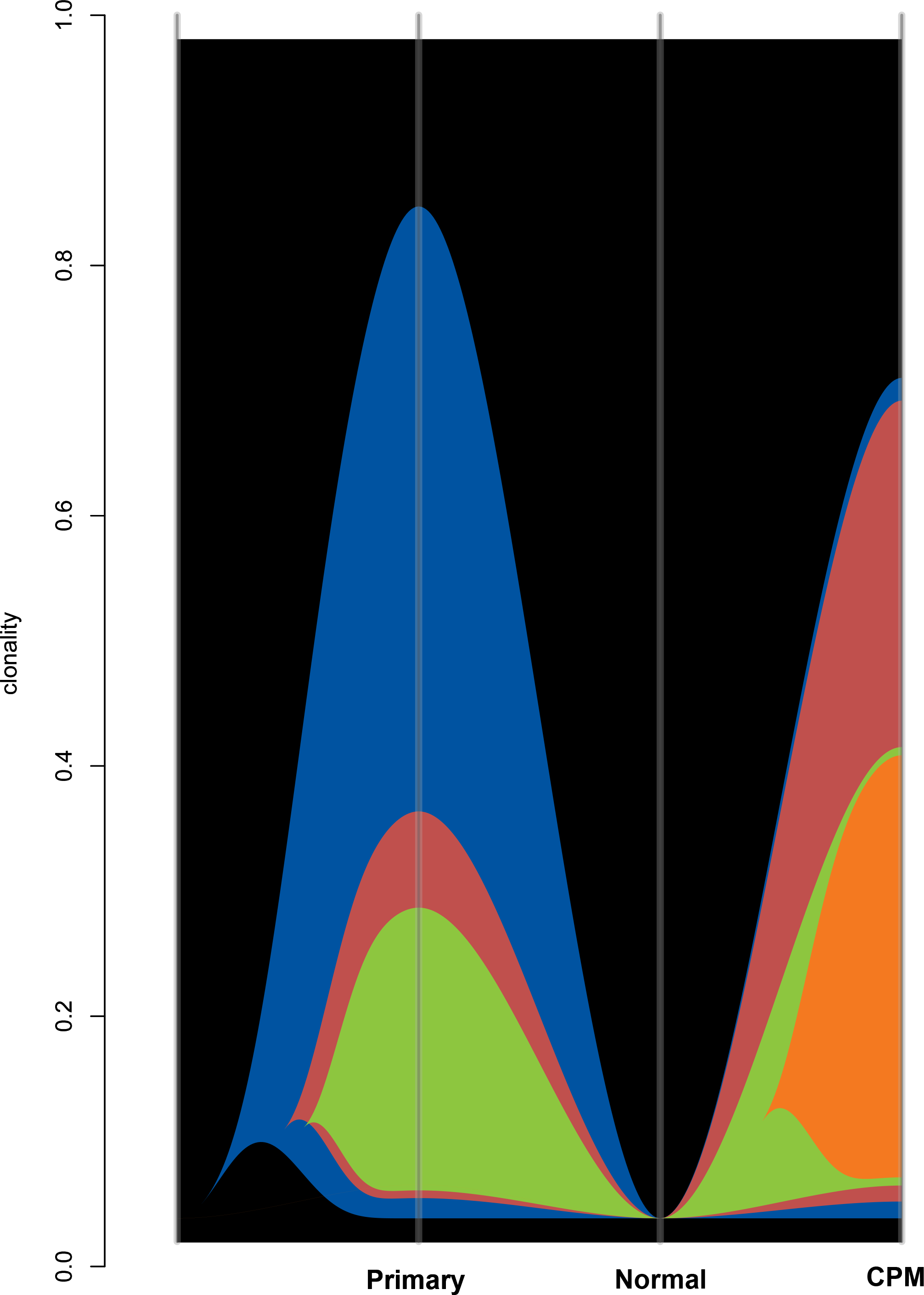


Supplementary table 1: KEGG pathway enrichment analysis, primary CRC vs. matched metachronous CPM and non-responders vs. responders to CRS & HIPEC (p-value < 0.05)

| **Gene set** | **Class** | **KEGG pathway** | **Enrichment score** | **P-value** |
| --- | --- | --- | --- | --- |
| **Primary CRC vs. matched CPM** | | | | |
| hsa04810 | Cellular Processes; Cell motility | Regulation of actin cytoskeleton | 4.19 | 0.02 |
| hsa05206 | Human Diseases; Cancers: Overview | MicroRNAs in cancer | 3.44 | 0.03 |
| hsa05222 | 'Human Diseases; Cancers: Specific types | Small cell lung cancer | 3.43 | 0.03 |
| hsa04672 | Organismal Systems; Immune system | Intestinal immune network for IgA production | 3.30 | 0.04 |
| hsa04670 | Organismal Systems; Immune system | Leukocyte transendothelial migration | 3.22 | 0.04 |
| **Non-responders vs. responders to CRS & HIPEC** | | | | |
| hsa04144 | Cellular Processes; Transport and catabolism | Endocytosis | 11.67 | 8.56 x10-6 |
| hsa00130 | Metabolism; Metabolism of cofactors and vitamins | Ubiquinone and other terpenoid-quinone biosynthesis | 5.07 | 6.28 x10-3 |
| hsa05100 | Human Diseases; Infectious diseases: Bacterial | Bacterial invasion of epithelial cells | 4.76 | 8.56 x10-3 |
| hsa05110 | Human Diseases; Infectious diseases: Bacterial | Vibrio cholerae infection | 4.14 | 0.02 |
| hsa04145 | Cellular Processes; Transport and catabolism | Phagosome | 3.92 | 0.02 |
| hsa05165 | Human Diseases; Infectious diseases: Viral | Human papillomavirus infection | 3.85 | 0.02 |
| hsa00561 | Metabolism; Lipid metabolism | Glycerolipid metabolism | 3.64 | 0.03 |
| hsa04966 | Organismal Systems; Excretory system | Collecting duct acid secretion | 3.63 | 0.03 |
| hsa00230 | Metabolism; Nucleotide metabolism | Purine metabolism | 3.37 | 0.03 |
| hsa05120 | Human Diseases; Infectious diseases: Bacterial | Epithelial cell signalling in Helicobacter pylori infection | 3.31 | 0.04 |
| hsa00020 | Metabolism; Carbohydrate metabolism | Citrate cycle (TCA cycle) | 3.20 | 0.04 |
| hsa01230 | Biosynthesis of amino acids | Biosynthesis of amino acids | 3.20 | 0.04 |
| hsa04520 | Cellular Processes; Cellular community – eukaryotes | Adherens junction | 3.13 | 0.04 |
| hsa03020 | Genetic Information Processing; Transcription | RNA polymerase | 11.67 | 8.56 x10-6 |
| hsa04068 | Environmental Information Processing; Signal transduction | FoxO signalling pathway | 5.07 | 6.28 x10-3 |

Supplementary table 2: The top 10 genes with significantly altered expression (FDR<0.1) in CPM non-responders to CRS & HIPEC

| **Rank** | **Gene name** | **Description** | **Fold change** | **FDR p value** |
| --- | --- | --- | --- | --- |
| **Genes with reduced expression in non-responders vs. responders to CRS & HIPEC** | | | | |
| 1 | KIAA0319L | Protein coding gene | 1.30 | 2.89 x10-03 |
| 2 | CORO7 | F-actin regulator | 1.16 | 7.36 x10-04 |
| 3 | DIP2C | Disco interacting factor | 1.13 | 1.90 x10-04 |
| 4 | STK38 | Serine/threonine kinase | 1.05 | 6.38 x10-05 |
| 5 | CLINT1 | Clathrin Interacting factor | 1.03 | 2.73 x10-03 |
| **Genes with increased expression in non-responders vs. responders to CRS & HIPEC** | | | | |
| 1 | VEZF1 | Endothelial cell specific transcription factor | -10.40 | 3.76 x10-05 |
| 2 | CEACAM1 | Carcinoembryonic antigen immunoglobulin | -8.27 | 1.83 x10-03 |
| 3 | BCYRN1 | Small non-messenger RNA | -7.96 | 7.50 x10-05 |
| 4 | RP11-192H23.5 | Long non-coding RNA | -6.22 | 2.38 x10-04 |
| 5 | RELB | Proto-Oncogene | -6.17 | 9.50 x10-04 |
| 6 | DCAF12 | Apoptosis regulator | -6.17 | 1.36 x10-05 |
| 7 | POLR3GL | RNA polymerase | -6.05 | 1.48 x10-03 |
| 8 | FEM1C | Protein coding gene | -5.69 | 1.87 x10-03 |
| 9 | AXIN1 | Negative Wnt pathway regulator | -5.42 | 1.67 x10-04 |
| 10 | KHDRBS3 | RNA-binding protein regulates alternative splicing | -5.01 | 1.23 x10-03 |

Supplementary table 3: The top 10 differentially methylated regions, (DMRs), CPM vs. primary CRC and non-responders vs. responders to CRS & HIPEC

| **Chr** | **Start**  **(bp)** | **End**  **(bp)** | **Length**  **(bp)** | **DMR**  **p-value** | **Located**  **Gene** | **UCSC CpG**  **island** |
| --- | --- | --- | --- | --- | --- | --- |
| **CPM vs. primary CRC** | | | | | | |
| chr11 | 2160904 | 2161586 | 682 | 0.002 | IGF2 | 302 |
| chr19 | 37157318 | 37157945 | 627 | 0.003 | ZNF461 | 39 |
| chr15 | 79383167 | 79383980 | 813 | 0.003 | RASGRF1 | 195 |
| chr18 | 35145983 | 35147090 | 1107 | 0.003 | CELF4 | 196 |
| chr19 | 58609269 | 58609987 | 718 | 0.003 | ZSCAN18 | 61 |
| chr13 | 78493229 | 78493712 | 483 | 0.003 | EDNRB | 70 |
| chr6 | 28602543 | 28603027 | 484 | 0.004 | ZBED9 | 32 |
| chr5 | 135415693 | 135416613 | 920 | 0.005 | VTRNA2-1 | 24 |
| chr19 | 58458572 | 58459358 | 786 | 0.005 | ZNF256 | 52 |
| chr5 | 38258007 | 38259243 | 1236 | 0.008 | EGFLAM | 108 |
| **Non-responders vs. responders to CRS & HIPEC** | | | | | | |
| chr10 | 134600357 | 134600919 | 562 | 0.007 | NKX6-2 | 569 |
| chr12 | 133464496 | 133464737 | 473 | 0.003 | CHFR | 91 |
| chr10 | 8096311 | 8096991 | 680 | 0.006 | NA | 509 |
| chr20 | 30639816 | 30640256 | 440 | 0.009 | HCK | 71 |
| chr16 | 54972078 | 54973579 | 1501 | 0.015 | NA | 179 |
| chr11 | 2397201 | 2397655 | 454 | 0.016 | BC019904 | NA |

Supplementary table 4 :The top 10 CNAs, CPM vs. primary CRC and non-responders vs. responders to CRS & HIPEC

| **Chr** | **Start** | **End** | **Type** | **Count** | **Score** | **GW_P** | **Gene** |
| --- | --- | --- | --- | --- | --- | --- | --- |
| **CPM vs. primary CRC** | | | | | | | |
| chr11 | Gain | 3817692 | 3818253 | 22 | 0.91 | 2.78 X10-07 | NUP98 |
| chr11 | Gain | 3825449 | 4629195 | 22 | 0.91 | 2.78 X10-07 | RRM1, TRIM21 |
| chr11 | Gain | 4629529 | 5704417 | 22 | 0.91 | 2.78 X10-07 | MMP26, UBQLN3,  TRIM5, 6 |
| chr11 | Gain | 5705995 | 5705995 | 22 | 0.91 | 2.78 X10-07 | TRIM5 |
| chr11 | Gain | 5712109 | 6518263 | 22 | 0.91 | 2.78 X10-07 | TRIM3, 5, 22, FAM160A2, CCKBR, SMPD1, APBB1, ARFIP2 |
| chr11 | Gain | 6518907 | 6650480 | 22 | 0.91 | 2.78 X10-07 | DNHD1, ILK, TPP1, TAF10, RRP8 |
| chr16 | Gain | 56635680 | 56645731 | 22 | 0.91 | 2.78 X10-07 | MT1A, 2A, 3, 4 |
| chr16 | Gain | 57333722 | 57335017 | 22 | 0.91 | 2.78 X10-07 | TRNALeu |
| chr16 | Gain | 57495650 | 57503371 | 22 | 0.91 | 2.78 X10-07 | POLR2C |
| chr16 | Gain | 71842906 | 71843647 | 22 | 0.91 | 2.78 X10-07 | AP1G1 |
| **Non-responders vs. responders to CRS & HIPEC** | | | | | | | |
| chr3 | 6902689 | 6905031 | Loss | 12 | 1 | 2.22 X10-16 | GRM7 |
| chr3 | 16554466 | 16554910 | Loss | 12 | 1 | 2.22 X10-16 | RFTN1 |
| chr3 | 36985697 | 36986697 | Loss | 12 | 1 | 2.22 X10-16 | TRANK1 |
| chr3 | 43020703 | 43022351 | Loss | 12 | 1 | 2.22 X10-16 | FAM198A |
| chr3 | 44596684 | 44596846 | Loss | 12 | 1 | 2.22 X10-16 | ZKSCAN7 |
| chr3 | 46939978 | 46940439 | Loss | 12 | 1 | 2.22 X10-16 | PTH1R |
| chr4 | 25657365 | 25657628 | Loss | 12 | 1 | 2.22 X10-16 | SLC34A2 |
| chr4 | 57975793 | 57976944 | Loss | 12 | 1 | 2.22 X10-16 | IGFBP7 |
| chr4 | 81187125 | 81188051 | Loss | 12 | 1 | 2.22 X10-16 | FGF5 |
| chr4 | 81951953 | 81952024 | Loss | 12 | 1 | 2.22 X10-16 | BMP3 |

*Chr, chromosomes, Type CNA, Start, start position of the CNA, End, end position of the CNA, count, number of samples carrying this type of event at this location, score, proportion of samples carrying this type of event at this location, GW_P, Poisson-binomial test for overlap of event across individuals at this location given proportion chromosome affected, Gene – gene encoded by this portion of the genome.*
